# Experimental setup for high-resolution characterization of crystal optics for coherent X-ray beam applications

**DOI:** 10.1107/S1600576722010998

**Published:** 2023-02-01

**Authors:** Aliaksei Halavanau, Rachel Margraf, River Robles, James MacArthur, Zhengxian Qu, Gabriel Marcus, Juhao Wu, Takahiro Sato, Diling Zhu, Christopher J. Takacs, Ross Arthur, Olga Kraynis, Bart Johnson, Thomas Rabedeau

**Affiliations:** a SLAC National Accelerator Laboratory, Stanford University, Menlo Park, CA 94025, USA; b Stanford University, Menlo Park, CA 94025, USA; Oak Ridge National Laboratory, USA; North Carolina State University, USA

**Keywords:** Bragg crystals, rocking curve imaging, Darwin width, cavity-based X-ray sources

## Abstract

Stanford Synchrotron Radiation Lightsource serves a wide scientific community with its variety of X-ray capabilities. Recently, a wiggler X-ray source located at beamline 10-2 has been employed to perform high-resolution rocking curve imaging (RCI) of diamond and silicon crystals. X-ray RCI is invaluable for the development of upcoming cavity-based X-ray sources at SLAC, including the cavity-based X-ray free-electron laser and X-ray laser oscillator. In this paper, the RCI apparatus is described and experimental results are provided to validate its design. Future improvements of the setup are also discussed.

## Abstract 2

The article presents the rocking curve imaging instrument at Stanford Synchrotron Radiation Lightsource beamline 10-2, which is used to characterize diamond and silicon crystals for Bragg optics applications.

## 1. Introduction

Cavity-based X-ray sources employing Bragg crystals, *e.g.* the cavity-based X-ray free-electron laser (CBXFEL), X-ray laser oscillator (XLO) and X-ray free-electron laser oscillator (XFELO) (Marcus *et al.*, 2020 ▸; Halavanau *et al.*, 2020 ▸; Kim *et al.*, 2008 ▸), are promising new instruments designed for obtaining better longitudinal coherence in the hard X-ray regime. In these projects, Bragg crystals define the spectral bandwidth and radiation wavefront properties, and are generally required to be of the highest optical quality. Synthetic diamond and silicon crystals are a common choice due to their high structural uniformity, thermal conductivity and excellent reflectivity in the hard X-ray range.

Below we describe an experimental setup for crystal quality measurement and qualification, based on the commonly known technique of rocking curve imaging (RCI), at the Stanford Synchrotron Radiation Lightsource (SSRL) 10-2 wiggler beamline. Extensive RCI studies of diamond and silicon crystals have been previously conducted at the European Synchrotron Radiation Facility (ESRF, France), Advanced Photon Source (APS, USA) 1BM and SPring-8 (RIKEN, Japan) 1 km-long beamlines, and our experimental setup complements these experiments (Lübbert *et al.*, 2000 ▸; Tamasaku *et al.*, 2001 ▸; Tamasaku *et al.*, 2005 ▸; Macrander *et al.*, 2005 ▸; Stoupin *et al.*, 2016*a* ▸; Macrander *et al.*, 2019 ▸; Pradhan *et al.*, 2020 ▸).

According to the theory of dynamic diffraction, a flat perfect crystal with the reciprocal lattice vector **H** transforms an incident electric field *E*_0_ in Bragg geometry as (Zachariasen & Hill, 1946 ▸; Batterman & Cole, 1964 ▸; Shvyd’ko & Lindberg, 2012 ▸; Lindberg & Shvyd’ko, 2012 ▸) 

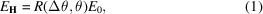

where *R*(Δθ, θ) is the complex reflectivity coefficient (for a flat perfect crystal) given by 

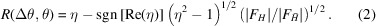

The parameter η is given by 

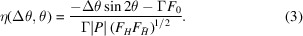

Here *F*_0_, *F*_*H*_ are the components of the structure factor, θ is the Bragg angle at the central photon energy ω_0_, *P* = 1 for σ polarization and 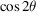 for π polarization, 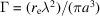 , *r*_e_ is the classical radius of the electron, and *a* is the lattice constant. We note that in cavity-based XFELs crystals with almost no miscut angles are typically employed, and therefore we consider the asymmetry parameter to be |*b*| ≃ 1. Structure factors can be calculated from the atomic scattering factors and are well tabulated for a wide range of photon energies [see *e.g.* Sutter *et al.* (2014 ▸), Cromer & Mann (1968 ▸) and Chantler (1995 ▸)]. The profile of |*R*(Δθ)|^2^ for a fixed photon energy ω_0_ is also known as the rocking curve and can be experimentally measured.

**Figure 1:**
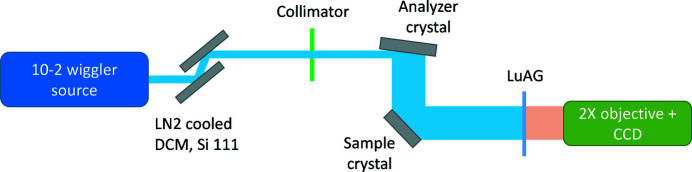
Experimental layout (not to scale) of the RCI setup.

**Figure 2:**
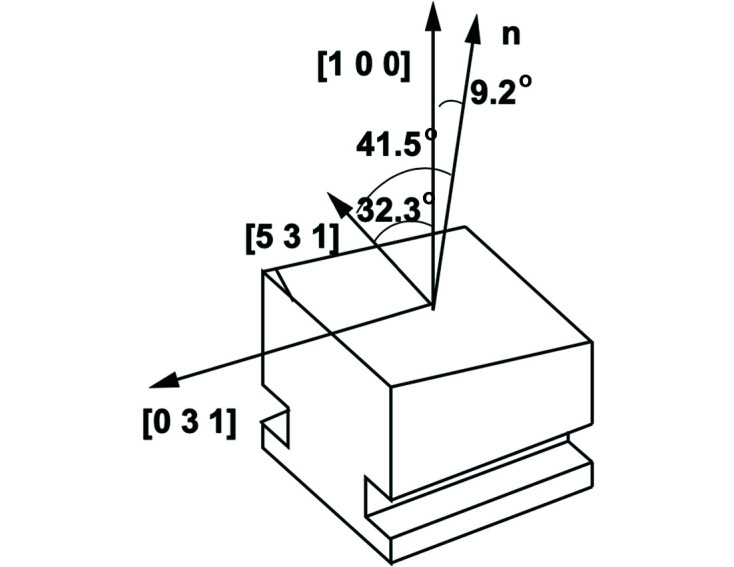
Design of an asymmetric Si(531) analyzer (collimator) crystal for RCI characterization of the C*(400) reflection in Bragg geometry.

In order to fully illuminate the sample surface with a narrow angular divergence beam, we used an asymmetric analyzer (collimator) crystal, in a non-dispersive geometry (see Fig. 1 ▸). This crystal was made out of highest diffraction grade silicon, due to its nearly perfectly periodic defect-free crystal structure. The main cut of the crystal is defined by a matching condition to have a value of *d* spacing as close as possible to that of the sample. For example, lattice constant of diamond is *a*_C_ = 3.567 Å, that of silicon is *a*_Si_ = 5.431 Å, and the *d* spacing can be calculated from *d* = [*a* ^2^/(*h* ^2^ + *k* ^2^ + *l* ^2^)]^1/2^. The narrowest rocking curve will be observed when the analyzer’s Miller indices minimize the expression 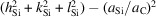 × 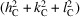 . Thus, a diamond C*(400) sample requires an Si(531) collimator. For more combinations of diamond reflections and corresponding analyzers, we refer the reader to Table 1 ▸. In addition, the analyzer (collimator) crystal was cut asymmetrically, according to the relation (Stoupin *et al.*, 2016*b* ▸) 

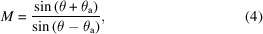

where θ is the Bragg angle, θ_a_ is the asymmetry angle and *M* is the magnification ratio. In our setup we used the ratio of *M* ≃ 25. An example design of an asymmetric Si(531) collimator is shown in Fig. 2 ▸. We also note that the process of RCI can be visually described with the help of DuMond diagrams (DuMond, 1937 ▸; Shvyd’ko, 2004 ▸)

**Figure 3:**
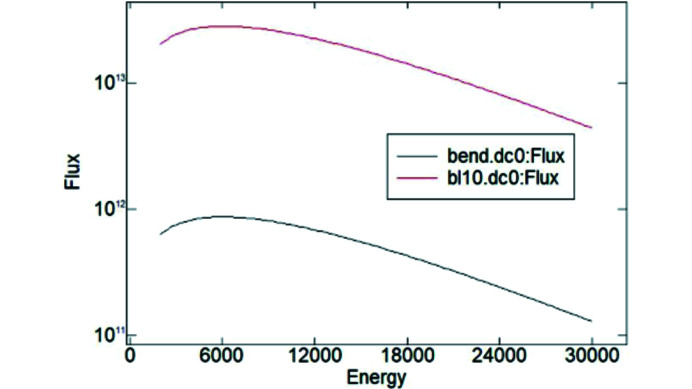
Spectral flux of the SSRL bending magnet and beamline 10-2 wiggler as a function of photon energy.

The high average brightness of the 15 period 10-2 wiggler results in a 30-fold increase in flux, compared with a single SSRL bending magnet (see Fig. 3 ▸). High photon flux at the photon energies of interest allows for ≤1 s exposure times per angular point. Hence the entire rocking curve is typically imaged in 1–2 min.

The last fact enables iterative, rapid studies of crystal mounting, clamping, bending *etc.*, which are invaluable for applications in X-ray cavities and monochromators. Our setup also offers the possibility of Laue topography, where the expanded beam from the collimator is apertured with a thin slit and diffracted through the sample in Laue geometry. We will report the Laue topography experimental results in a separate paper.

## 2. Experimental setup

**Figure 4:**
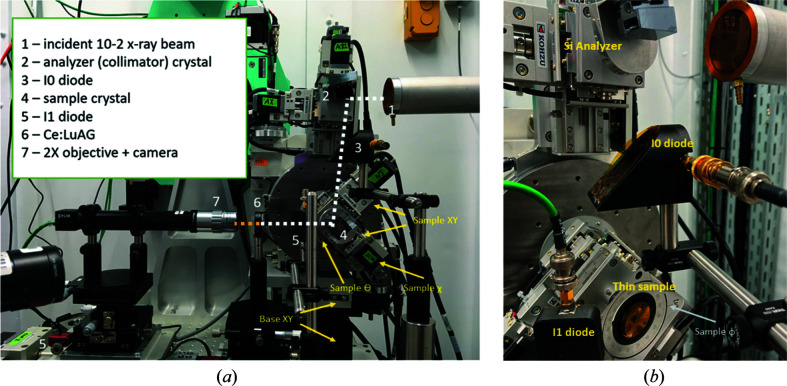
Photograph of the experimental setup and corresponding annotation (*a*). A close-up photograph of the configuration with silicon analyzer and thin diamond sample (*b*).

SSRL 10-2 wiggler beamline is operated in the unfocused configuration with an Si(111) liquid-nitrogen-cooled double-crystal monochromator (DCM). The DCM can be tuned to a desired photon wavelength. The combined motion stack is shown in Fig. 4 ▸. In brief, we use a θ–2θ RA-2021 ▸ stage (Kohzu) mounted on top of the vertical and horizontal Kohzu translation stages (base XY) to position the analyzer (collimator) crystal into the incoming beam. The 2θ arm of the RA-2021 ▸ is used for the collimator alignment. An XY/tip–tilt stack of stages (Kohzu) is then used to set the final collimator position and out-of-plane angle (χ angle). We acknowledge that in this configuration the canonical θ and χ axes of rotation of the analyzer crystal do not strictly coincide with the experimental axes, but we minimize these errors through iterative alignment.

Following the analyzer, the X-ray beam footprint overfills the sample. Thick samples are mounted on an Al platform, while thin samples are typically mounted in a Kapton foil sandwich (see Fig. 4 ▸). The foil is electrostatically pre-charged. This mounting method has been proven to have minimal angular drifts on the order of 2 µrad for thin samples, while being very robust for thicker samples. We can replace the Kapton sandwich with a physical clamp, a gravity mount or other types of mounts. The samples are scanned using θ motion of the RA-2021 ▸.

A 20 µm thick Ce:LuAG screen converts the diffracted X-rays into the optical signal, and a 2× magnification objective is used to transport the optical signal onto the CMOS sensor (FLIR). Each camera image is acquired synchronously with the RA-2021 motion. The final image resolution is about 1.7 µm.

## 3. Experimental results

**Figure 5:**
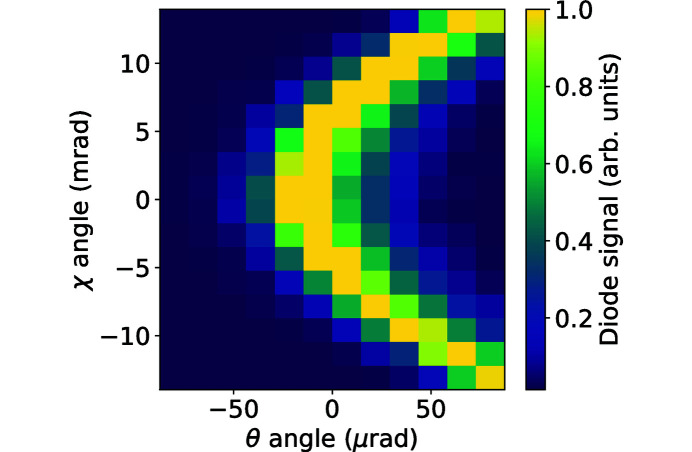
An example 2D χ–θ scan in C*(400) Bragg reflection geometry. The optimized sample position corresponds to the origin point on the plot.

Initial alignment is performed with a high-sensitivity PIPS photodiode. After the Bragg reflection off the sample is established, we proceed with the χ-scan routine. In this routine we optimize the χ angle of the sample, to align the reflecting crystal planes parallel to the θ-stage rotation axis. This is an important step to minimize geometrical errors, resulting in observed unphysical horizontal strain gradient. Note that, when the χ angle is varied, the θ angle should also be scanned, and thus the χ-scan procedure is a 2D scan. An example χ scan is shown in Fig. 5 ▸ [see also Bowen & Tanner (1998 ▸)].

After the χ scan is complete, we position the sample at the leftmost point on the χ(θ) curve, and proceed with rocking the sample by a 1D θ scan. As a result of this scan, we arrive with a set of sample images, taken at different θ positions. These images are then stacked in a 3D array, with dimensions of (*xy*θ). A Gaussian curve is fitted to each point in (*xy*) as a function of θ, thus yielding the Darwin curve width and its center position. These values can then be plotted as a function of (*xy*) coordinates, generating rocking curve maps of the sample for further analysis. A similar procedure has been recently outlined by Pradhan *et al.* (2020 ▸). Our method, albeit simple, allows for quick and accurate assessment of the strain field gradients, dislocations, stacking faults, inclusions and other defects in the sample for a given reflection.

### 3.1. Silicon crystals

**Figure 6:**
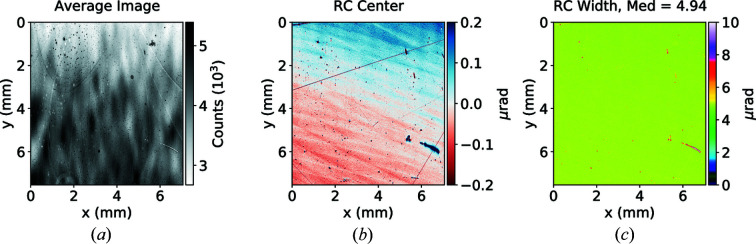
Rocking curve imaging summary for an Si reference crystal in the (531) Bragg reflection. Plotted are the average obtained image (*a*), variation of rocking curve center (*b*) and variation of rocking curve width (*c*) along the crystal surface. Total acquisition time was 44 s.

Highest-grade silicon crystals typically have excellent X-ray diffraction properties. Therefore we started with measuring flat perfect Si(531) in the Bragg geometry. We have used super-polished Si crystals with a surface roughness of less than 5 nm. To measure a large silicon crystal, we replaced our Kapton foil sandwich and ϕ stage with a metal mount which gently clamped the base of the thick silicon block. We observe a vertical strain gradient of about ±0.2 µrad, which is likely attributable to the small amount of lattice strain or wavefront curvature of the wiggler source (see Fig. 6 ▸). The initial intensity fluctuations in the wavefront are almost negligible in the RCI analysis since they are the same for each θ point and are averaged out during fitting. Fig. 6 ▸(*a*) shows wavefront distortions that are almost invisible in Fig. 6 ▸(*b*). However, to mitigate incident waveform distortions, the beamline must contain highly polished vacuum windows. In our case, highly polished Be windows were installed in the beamline. The total Kapton foil thickness in the entire X-ray path was about 200 µm.

### 3.2. Diamond crystals

**Figure 7:**
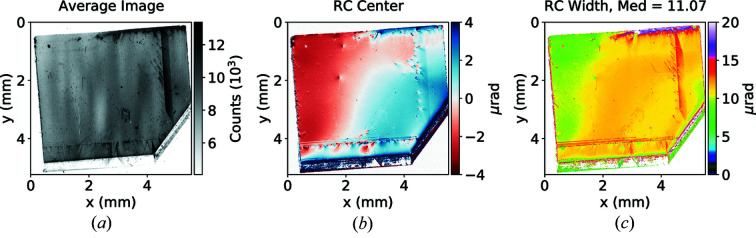
Rocking curve imaging summary for a C*(400) Bragg reflection. Plotted are the average obtained image (*a*), variation of rocking curve center (*b*) and variation of rocking curve width (*c*) along the crystal surface. Total acquisition time was 110 s.

We then proceeded with characterizing high-pressure high-temperature (HPHT) type II-a diamond samples. These samples tend to have very high uniformity of the crystal structure, although not as good as the highest-grade silicon. A typical rocking curve of high-quality HPHT type II-a diamond is shown in Fig. 7 ▸.

**Figure 8:**
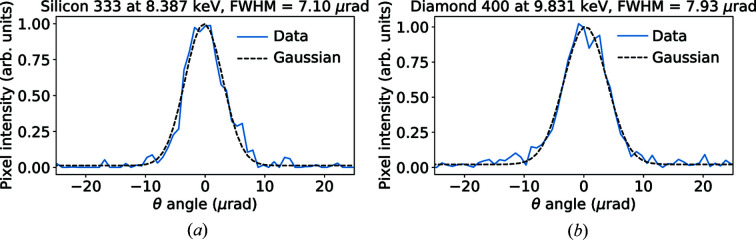
Single-pixel rocking curves for Si(333) (*a*) and C*(400) (*b*) Bragg reflections. Total acquisition time was 126 s for the 333 reflection and 90 s for the 400 reflection.

We also provide an example of single-pixel rocking curves fitted with a Gaussian (see Fig. 8 ▸). Note that in both cases the fit quality is quite good, giving credibility to our approach.

**Figure 9:**
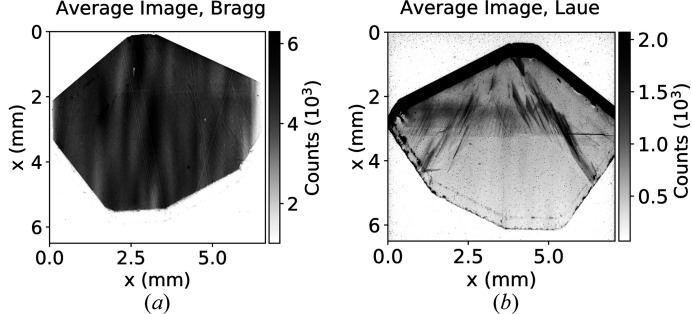
Comparison of the average X-ray image obtained in C*(220) Bragg geometry (*a*) and C*(400) Laue geometry (*b*) for the same sample. Total acquisition time was 110 s in Bragg geometry and 192 s in Laue geometry.

Part of the crystal characterization process is also the analysis of surface and bulk volume defects. The latter can be assessed in a single shot by the white-beam topography method (Tuomi *et al.*, 1974 ▸). In our setup, although we do not have white-beam capability, we can partially characterize the crystal volume by measuring Bragg reflections in Laue geometry orthogonal to their Bragg geometry counterparts. For instance, for (110) face-cut crystals we register the C*(220) Bragg reflection and the C*(400) Bragg reflection in Laue geometry (see Fig. 9 ▸).

**Figure 10:**
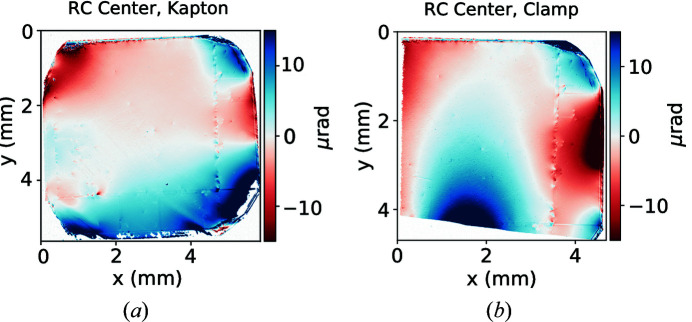
Comparison of the rocking curve center variation for the same crystal held by a Kapton sandwich (*a*) and by a physical clamp (*b*). Total acquisition time was 256 s for the unclamped and 176 s for the clamped case.

An important process in cavity-based XFEL experiments is diamond clamping. During clamping, the mechanical forces introduce strain fields that may propagate in the region of interest, and alter the diffraction properties of the crystal (see Fig. 10 ▸). To avoid that, a strain relief cut can be introduced (Pradhan *et al.*, 2020 ▸).

## 4. Summary

We have successfully built and commissioned a high-resolution rocking curve imaging setup for diamond and silicon crystals. Our setup utilizes the SSRL beamline 10-2 wiggler source, a double-crystal silicon monochromator and an asymmetric silicon crystal analyzer. We specifically optimized the time of a single RCI measurement to allow for rapid studies of crystal holders and potentially cryo-cooling techniques. Our setup can be adapted for other types of Bragg mirrors, such as different types of crystals, thin films *etc*. In the future, we envision expanding our setup to different reflection geometries and X-ray tomography.

## Figures and Tables

**Table 1:** Accessible diamond Bragg reflections (at θ_B_ = 45.0°), corresponding silicon analyzers, characteristic photon energies, and Darwin curve FWHM (σ polarization), in our RCI setup (calculated with the *XOP* program; del Rio & Dejus, 2011 ▸)

C*	Si	θ_a_	ω (keV)	ΔΘ (μrad)
(220)	(331)	43.70	6.95	20.7
(400)	(531)	41.38	9.83	8.2
(333)	(800)	43.64	12.77	3.0
(440)	(555)	43.30	13.90	3.4
